# Homecoming: rewinding the reductive evolution of the chloroplast genome for increasing crop yields

**DOI:** 10.1038/s41467-021-26975-5

**Published:** 2021-11-18

**Authors:** Briardo Llorente, María Eugenia Segretin, Estefanía Giannini, Celina Lobais, Marcelo E. Juárez, Ian T. Paulsen, Nicolás E. Blanco

**Affiliations:** 1 ARC Center of Excellence in Synthetic Biology, Department of Molecular Sciences, Macquarie University, Sydney, NSW 2109 Australia; 2 Laboratorio de Biotecnología Vegetal, Instituto de Investigaciones en Ingeniería Genética y Biología Molecular “Dr. Héctor N. Torres” (INGEBI-CONICET), (C1428ADN), Ciudad Autónoma de Buenos Aires, Buenos Aires, Argentina; 3 Departamento de Fisiología, Biología Molecular y Celular, Facultad de Ciencias Exactas y Naturales, Universidad de Buenos Aires, (C1428EGA), Ciudad Autónoma de Buenos Aires, Buenos Aires, Argentina; 4 Center of Photosynthetic and Biochemical Studies (CEFOBI-CONICET), Faculty of Biochemical Science and Pharmacy, Rosario National University, S2002LRK Rosario, Argentina

**Keywords:** Synthetic biology, Agricultural genetics, Genetic engineering, Molecular engineering in plants

## Abstract

Developing more productive and sustainable crops will be essential to achieving food security in coming decades. A core process in plant evolution has been the transfer of chloroplast-encoded genes to the nuclear genome. We propose reverting this process as a new approach to improve plant disease resistance and photosynthesis in future crops.

## Crop yields and the reductive evolution of the chloroplast genome

Achieving sustainable food security is one of the most significant challenges of our time. This has to be accomplished in the face of global climate change causing increasingly severe environmental stresses and more frequent plant disease outbreaks affecting crop yields. Contemporary agriculture is also highly resource-intensive and places unprecedented pressure on the environment. Future crops will have to provide significant gains in yields while simultaneously reducing agriculture’s environmental impact in the general context of climate change^1^.

Chloroplasts are the plant organelles responsible for photosynthetic energy production and many other processes that underlie crop productivity, and therefore are primary targets for engineering agronomic traits. Chloroplasts and other plastids are thought to have originated through endosymbiotic evolution from a photosynthetic prokaryote that assimilated with a eukaryotic host more than a billion years ago. Over time, the genome of the prokaryotic endosymbiont underwent a reduction in size, mainly caused by the loss of genetic information that was dispensable in the context of endosymbiosis and the relocation of genes to the host nuclear genome^2^. Modern chloroplasts retain a relatively small functional genome called the plastome that typically harbors only 100–250 genes^3^. However, chloroplasts depend on thousands of proteins that derive from nucleus-encoded chloroplast genes (NECGs) and are imported following synthesis in the cytosol^4^. Accordingly, plants have evolved signaling networks that mediate nucleus−chloroplast bidirectional communication and mechanisms that adjust the expression of NECGs in response to continually varying internal and external stimuli to sustain chloroplastic functions^5^.

Many plant pathogens cause disease by disrupting the chloroplast−nucleus communication to generate a favorable environment for proliferation, thus reducing crop productivity^6^. Furthermore, photosynthesis, the primary determinant of crop yield, is highly reliant on the communication between the chloroplast and the nucleus to continuously adapt to changing environmental conditions^7^. However, the chloroplast−nucleus communication entails intrinsic temporal and specificity constraints limiting photosynthetic efficiency and crop yield potential^8^. Here we assess the feasibility of bypassing the chloroplast−nucleus communication by relocating genes from the nuclear genome to the plastome as a new strategy for improving plant disease resistance and photosynthesis. We examine prospective strategies toward this end and discuss opportunities and challenges arising from exploring these approaches to develop more productive and sustainable crops.

## Improving plant disease resistance

Plant pathogens cause extensive global losses in yield and quality of agricultural production, posing a continuous threat to food security^9^. Today, crop disease management is based primarily on the application of chemical control agents. The most effective and sustainable alternative to the use of chemical control methods is implementing genetic disease resistance. However, one major challenge to the implementation of genetic disease resistance is to achieve long-lasting protection against pathogens, which typically adapt rapidly to overcome resistance.

**Fig. 1:**
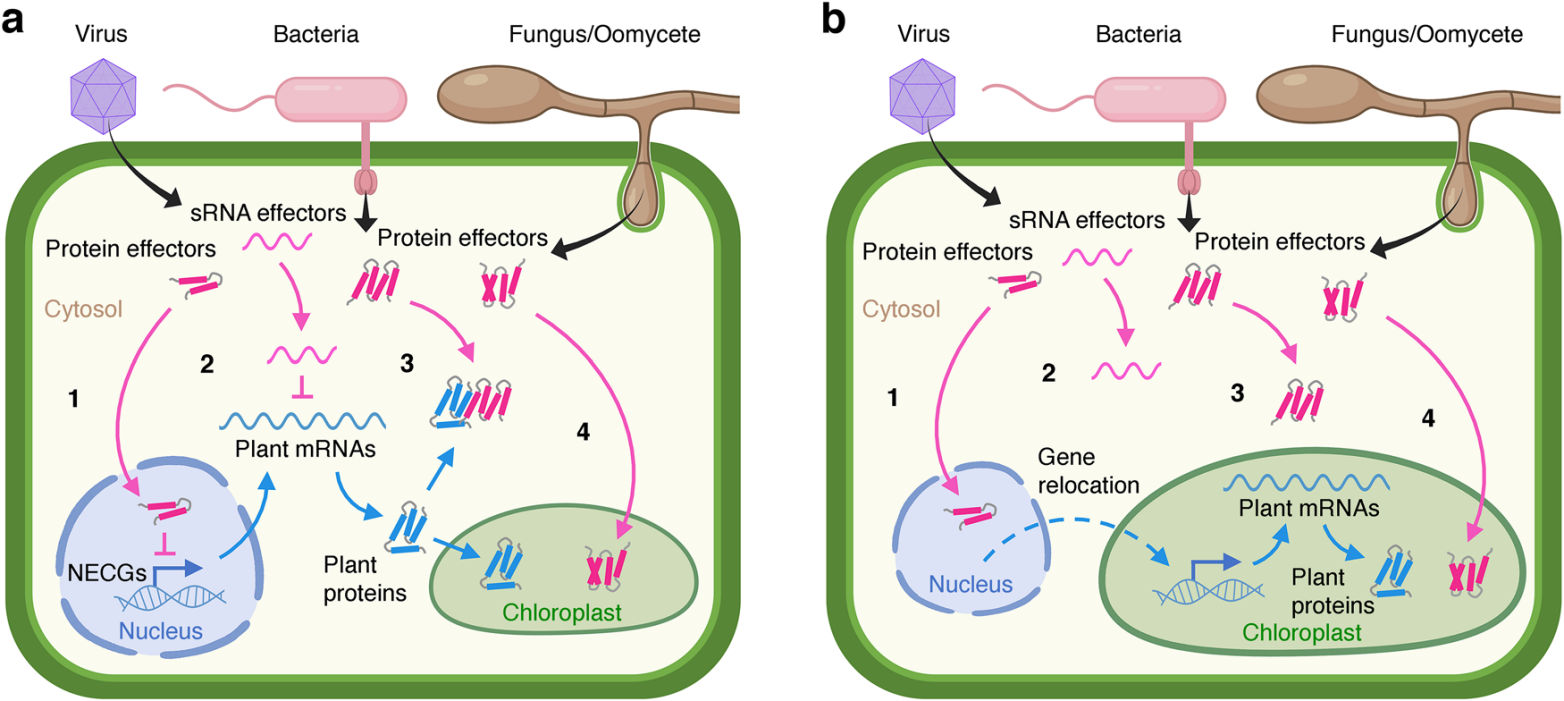
Relocating nuclear genes to the plastome for improving plant disease resistance. **a** Pathogens deploy protein and small RNA (sRNA) effectors (in pink) into plant cells that disrupt chloroplastic functions. Effectors can alter the expression of nucleus-encoded chloroplast genes—NECGs (1 and 2), inhibit the delivery of host proteins to the chloroplast (3), or translocate into the chloroplast to interfere directly with its functioning (4). **b** The relocation of NECGs targeted by pathogens’ effectors from the nuclear genome to the plastome would render effectors acting via 1, 2, and 3 ineffective.

To cause disease, pathogens deploy protein and small RNA (sRNA) effectors that enter the plant cell and modulate its physiology to promote infection. Many different plant pathogens, including viruses, bacteria, fungi, and oomycetes, have convergently evolved effectors that specifically interfere with chloroplastic functions. Pathogens accomplish this through at least three different strategies based on the deployment of effectors that alter the expression of NECGs, disrupt the delivery of proteins to the chloroplast, or translocate into the chloroplast to interfere directly with its functioning^10–13^ (Fig. 1a).

We reason that relocating to the plastome NECGs targeted by pathogens’ effectors would uncouple critical plant−pathogen interactions that pathogens have evolved to promote infection. Through this approach, it follows that it could be possible to confer increased resistance to disease because pathogens’ effectors would no longer be able to target plastome-relocated genes or their mRNA and protein products (Fig. 1b). Genes relocated to the plastome would continue to provide proteins to the chloroplast environment. However, we predict this would have an extremely disruptive impact on infection strategies established through a long history of evolutionary arms races between plants and pathogens, potentially generating an effective barrier to pathogens’ adaptation. The approach could also be expanded to relocate to the plastome multiple NECGs targeted by diverse pathogens to confer broad-spectrum disease resistance. Alternatively, broad-spectrum disease resistance could also be attained by transferring to the plastome fewer ‘hotspot’ NECGs identified to be common targets to many different pathogens. We also anticipate that the effectiveness of the proposed resistance mechanism could be increased by relocating to the plastome several NECGs targeted by a pathogen, hence causing multiple effectors to become ineffective and creating an even more significant obstacle to pathogens’ adaptation.

While the relocation of genes from the nucleus to the chloroplast could enhance plant disease resistance by evading pathogens’ effectors that alter the expression of NECGs or disrupt protein delivery to the chloroplast, we expect that it would not be effective against effectors that translocate into the chloroplast. Nevertheless, not all pathogens produce effectors that localize to chloroplasts, and we speculate that the proposed approach would still likely reduce the severity of infections caused by pathogens that deploy a mixture of effectors acting inside and outside the chloroplast. In support of this hypothesis, many previous works have shown that the alteration of just one single pathogen effector or plant target gene can be sufficient to significantly increase plant disease resistance^14,15^.

## Improving photosynthesis

The photosynthetic processes have not been evolutionarily optimized for the conditions and needs of modern agricultural food production or to cope with current changes in the global climate. Hence, improving photosynthesis has long been identified as a primary target with enormous potential to significantly enhance crop yields.

Even though photosynthesis takes place in chloroplasts, most genes participating in photosynthesis and associated processes are encoded by the nuclear genome^4^. Therefore, although chloroplasts have buffering mechanisms to adapt their photosynthetic metabolism to fluctuating conditions, the photosynthetic processes depend heavily on the communication between the chloroplast and the nucleus to adjust their functioning. However, this communication entails intrinsic constraints that can reduce photosynthetic efficiency. Chloroplast−nucleus communication is not instantaneous, and adaptive responses involving NECGs typically lag behind rapid and irregular fluctuations in environmental conditions (e.g., variations in light intensity and quality)^8^. The specificity of short-term photosynthetic responses is also limited by the confluence of multiple signaling pathways that integrate into the nucleus. Information from different sources (e.g., light receptors, metabolites concentrations, chloroplast redox status) have overlapping effects in the regulation of NECGs, resulting in balanced responses that are not always optimally adapted to rapidly changing environmental factors^7^. Responses orchestrated by the nucleus also have additional specificity limitations due to chloroplasts’ heterogeneity within photosynthetic cells, which typically harbor many chloroplasts. Signals derived from multiple chloroplasts are collectively integrated by the nucleus to change the expression of NECGs and generate equalized responses that likely affect all chloroplasts in a cell rather equally. However, because the functional and physiological states of all chloroplasts in a cell are not expected to be homogeneous, it is assumable that centralized nuclear responses would not be optimal for each chloroplast’s particular condition.

We argue that the plastome relocation of photosynthesis-related NECGs could be exploited to hasten adaptive responses adjusted to individual chloroplast’s specific requirements, thus optimizing photosynthetic performance in every single chloroplast. Although the relocation of NECGs to the plastome has previously been investigated for different research goals (e.g., to study electron fluxes along the photosynthetic electron transport chain^16^), we contemplate underexplored biotechnological potential to improve photosynthesis by conferring chloroplasts with more autonomy to adapt their photosynthetic functioning beyond nuclear control. More autonomous chloroplasts could individually adapt to changes more quickly and optimally to always keep photosynthesis functioning at the highest level of efficiency, something that can be argued is not possible when photosynthetic adaptations depend on nucleus-centralized responses.

## Developing synthetic adaptive gene expression regulation in chloroplasts

**Fig. 2:**
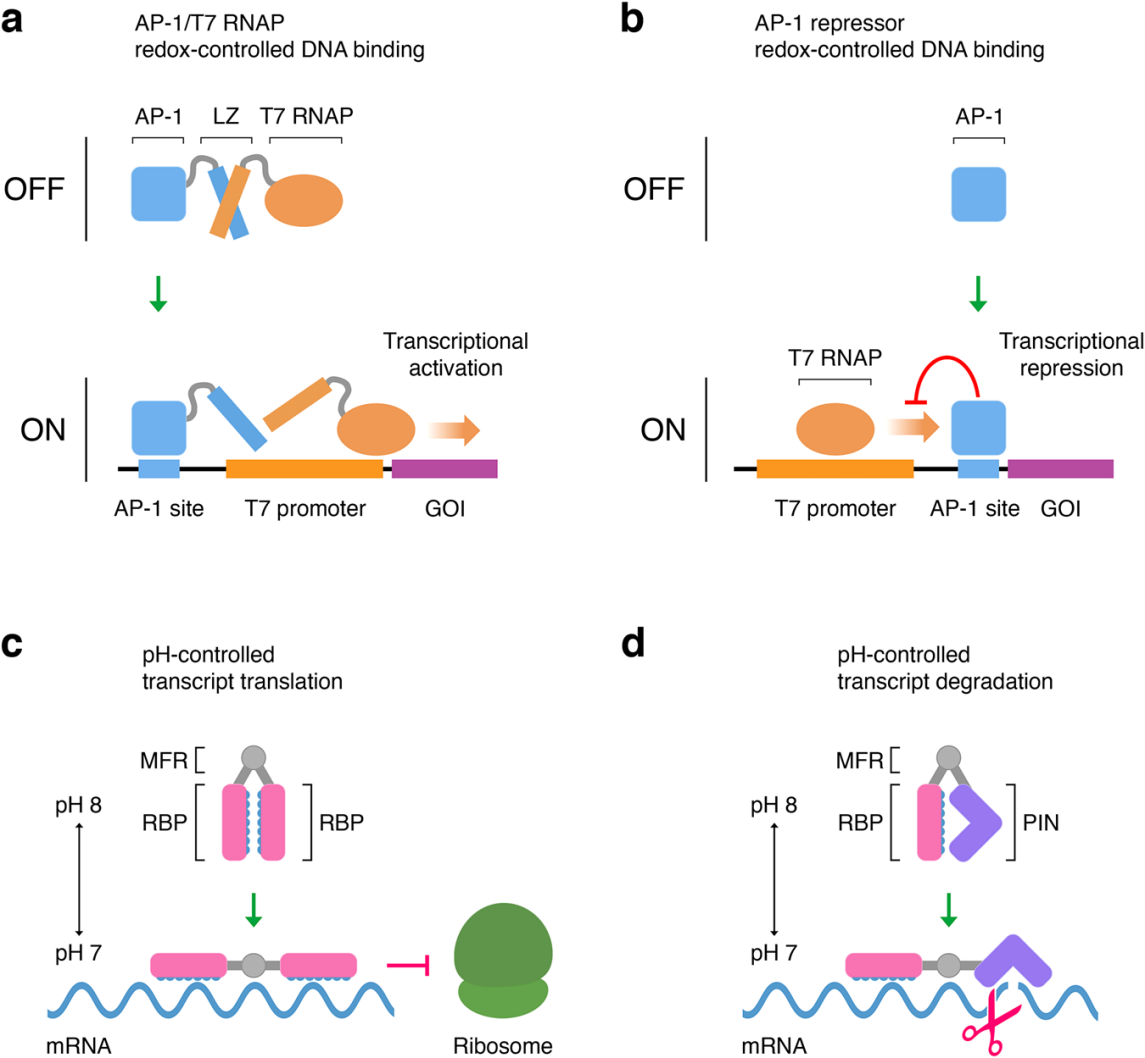
Prospective synthetic systems for engineering adaptive gene expression regulation in chloroplasts. **a** Design for a synthetic redox-controlled transcriptional activator system. The activator protein-1 (AP-1), tethered through leucine zipper domains (LZ) to the phage T7 RNA polymerase (T7 RNAP), binds to the DNA sequence AP-1 site in response to redox changes and recruits the AP-1/T7 RNAP complex to the T7 promoter. Transcription of the gene of interest (GOI) is initiated following promoter escape of the T7 RNAP. Tethering the AP-1 and T7 RNAP modules through LZ (instead of direct fusion) would enable T7 RNAP detachment, facilitating promoter escape that would result in higher transcriptional activation rates^17^. **b** Design for a synthetic redox-controlled transcriptional repressor system. The T7 promoter is placed upstream of the DNA sequence AP-1 site. AP-1 is recruited to the DNA sequence AP-1 site in a redox-dependent manner, blocking transcription by inhibiting promoter escape of the T7 RNAP. **c** By connecting two RNA-binding proteins (RBPs) through the mobile flap region (MFR) of the *Sporosarcina pasteurii* urease, it is possible to design synthetic post-transcriptional regulators with pH-dependent RBP-RNA-binding behavior. Changes in the stromal pH would switch the synthetic regulator from a closed inactive conformation to an open conformation that binds to specified transcripts to inhibit their translation. **d** By replacing one of the RBPs with a non-specific PIN ribonuclease, it is possible to design systems of analogous pH-dependent behavior that would cleave specified RNA targets when switched to an open conformation, leading to transcript degradation. In a closed conformation, the PIN ribonuclease is caged by the RBP region, and thus the systems remain inactive. In an open conformation, the RBP region can bind its target RNA while the PIN ribonuclease is uncaged to become active.

Realizing the vision of relocating nuclear genes to the plastome would also require engineering regulatory systems that sense stimuli and implement gene expression responses in chloroplasts. Adaptive plastome-autonomous gene expression regulation could be achieved by harnessing the chloroplast’s environmental sensing capacity. One way to accomplish this would be to engineer transcriptional regulation coupled to the chloroplast’s redox milieu. Environmental changes, such as fluctuating light conditions, alter the redox poise of multiple chloroplast systems (e.g., the plastoquinone pool, the thioredoxin system, the trans-thylakoid proton motive force)^5,8^. These principles could make it possible to build synthetic redox-responsive regulatory systems for controlling plastome-localized gene expression according to the chloroplast’s redox state. A vision of these systems is shown in Fig. 2a, b, which are examples of synthetic redox-responsive activator and repressor transcription factor designs based on the phage T7 RNA polymerase (T7 RNAP) and the activator protein-1 (AP-1). The T7 RNAP is readily programmable^17^ and functional in chloroplasts^18^, while AP-1 binds to cognate DNA (AP-1 site) under redox control^19^. Varying degrees of transcription activation and repression could be achieved by using T7 RNAP variants with different activities and by altering the affinity of the transcription factors to their binding sequences (e.g., by using T7 RNAP variants with altered specificity and promoters with different tandem copies of AP-1 sites). The fusion of AP-1 to other proteins further expands the possibilities to customize the architecture and activity of the transcriptional repressor system to engineer tuned repression of transcription initiation through steric hindrance of T7 RNAP binding to T7 promoter DNA.

Another conceivable strategy to develop plastome-autonomous gene expression regulation would be by building synthetic systems that respond dynamically to the chloroplast’s pH state. Chloroplasts manifest light-dependent changes in pH that are important for optimizing photosynthesis and carbon fixation^20^. While the chloroplast stroma has a neutral pH close to 7, upon illumination, the stromal pH is alkalized up to around 8. Conceptual synthetic pH-responsive regulatory systems are shown in Fig. 2c, d. These designs are based on the *Sporosarcina pasteurii* urease mobile flap region, which mediates pH-induced hinge-bending motion resulting in protein conformational changes^21^, and RNA-binding proteins (RBPs). RBPs, such as PPR (pentatricopeptide repeat) and PUF (Pumilio and FBF) proteins, can be programmed to bind with high specificity to RNA sequence targets^22^ and thus provide a foundation for building generic synthetic molecular systems to regulate gene expression in chloroplasts.

The development of these and other types of synthetic gene expression regulation systems (e.g., based on metabolite- or light-sensing riboswitches, CRISPR enzymes, and de novo protein design) would be a paradigm shift in chloroplast engineering, enabling rapid and optimized adaptive responses according to each chloroplast’s specific status.

## Opportunities, challenges, and future prospects

Over recent decades, an extensive molecular toolbox for plastome engineering has been developed. The relative simplicity, small size, and essentially prokaryotic nature of the plastome offer an advantageous genomic platform for engineering genetic processes of interest. Key plastome features include the lack of gene silencing, the possibility of arranging several genes into synthetic operons, and efficient gene targeting via homologous recombination^3^. Genetically engineered chloroplasts also have a remarkable protein metabolism flexibility^23^ and capacity to produce exceptionally high levels of heterologous proteins without causing deleterious plant phenotypes (e.g., >50% of total soluble proteins^24^). In addition, the prospect of installing fully synthetic plastomes into chloroplasts is indeed within reach now^25^. Thus, testing our concepts is technically feasible.

It is important to emphasize that the approach we propose here does not necessarily require, at least initially, deleting the nuclear copies of genes relocated to the plastome. Instead, it is conceivable that both nuclear and chloroplastic gene copies could coexist to sustain cell processes cooperatively. For instance, even if NECGs would remain targets of pathogens’ effectors, their plastome-relocated versions would still operate unaffected in the chloroplast to support plant functions during pathogen attack (Fig. 1). Furthermore, it is possible that having redundant genes located in the nuclear and chloroplast genomes functioning in parallel could synergistically enhance the basal activity of essential biochemical processes such as photosynthesis. Consistent with this idea, it was recently shown that the introduction of an additional nuclear copy of the *psbA* gene, encoding the D1 protein of photosystem II in the plastome, led to a significant increase in photosynthetic efficiency and crop yield under both normal and heat-stress conditions^26^.

One current limitation to exploring the plastome relocation of NECGs in crops is that the number of plant species whose plastomes can be engineered is still relatively small. However, agricultural relevant crops such as tomato, potato, lettuce, and tobacco, and the model plant *Arabidopsis thaliana* can be reliably transformed in their plastomes, and hence could be used as initial testbeds. Also, engineered plastomes can be horizontally transferred to currently non-transformable related species via grafting^3^. Another challenge may be the maximum number of NECGs that would need to be relocated to the plastome to improve agronomically important traits, since no more than a dozen genes have been simultaneously transformed into the chloroplast genome so far^27^. A further challenge would be achieving appropriate regulation on plastome-relocated genes to avoid potential negative consequences on plant growth and development. A good example would be if attempting the plastome relocation of carotenoid-related genes, which play functional roles in photosynthetic light-harvesting and photoprotection, but whose regulation would have to be carefully considered to mitigate unwanted carotenoid-derived effects on chloroplast development and functioning (e.g., loss of photosynthetic competence^28^). Adaptive plastome-autonomous gene expression regulation, in contrast to constitutive gene expression, would also reduce the energy and resource burden of expressing plastome-relocated genes. Hence, the development of synthetic chloroplastic gene expression systems, such as those discussed above (Fig. 2), would be critical for the engineering of nucleus-to-chloroplast gene relocations. Another foreseeable challenge could be encountered when relocating to the plastome NECGs involved in processes requiring coordination of multiple components, such as concerted assembly of multiprotein molecular machinery (e.g., photosystem II). The capacity to build synthetic operons in the plastome confers the possibility of addressing this challenge through engineering coordinated expression of multiple genes.

Radical plastome redesigns are now attainable, and we believe that there are justified biotechnological incentives beyond basic research to embark on ambitious plastome engineering endeavors. Motivated by the enormous potential of synthetic biology to revolutionize agriculture^29,30^, we have considered the relocation of genes from the nuclear genome to the plastome as a new way of improving plant disease resistance and photosynthesis. Current technologies should allow us to readily evaluate the conceptual approaches proposed here. Going forward, as plastome engineering techniques improve and become applicable to a broader range of species, it will be possible to explore more progressive approaches involving the relocation of increasing numbers of genes into the plastome and even fully synthetic plastomes in multiple plants. Ultimately, we envision that reverting the reductive genome evolution that shaped the plastome could open new opportunities to develop more productive and sustainable crops for the future.

### Reporting summary

Further information on research design is available in the Nature Research Reporting Summary linked to this article.

## Supplementary information

Reporting Summary

## References

[1] Tilman D, Balzer C, Hill J, Befort BL (2011). Global food demand and the sustainable intensification of agriculture. Proc. Natl Acad. Sci. USA.

[2] Timmis JN, Ayliffe MA, Huang CY, Martin W (2004). Endosymbiotic gene transfer: organelle genomes forge eukaryotic chromosomes. Nat. Rev. Genet..

[3] Boehm CR, Bock R (2019). Recent advances and current challenges in synthetic biology of the plastid genetic system and metabolism. Plant Physiol..

[4] Allen JF (2015). Why chloroplasts and mitochondria retain their own genomes and genetic systems: colocation for redox regulation of gene expression. Proc. Natl Acad. Sci. USA.

[5] Chan KX, Phua SY, Crisp P, McQuinn R, Pogson BJ (2016). Learning the languages of the chloroplast: retrograde signaling and beyond. Annu. Rev. Plant Biol..

[6] Sowden RG, Watson SJ, Jarvis P (2018). The role of chloroplasts in plant pathology. Essays Biochem..

[7] Unal D, Garcia-Caparros P, Kumar V, Dietz KJ (2020). Chloroplast-associated molecular patterns as concept for fine-tuned operational retrograde signalling. Philos. Trans. R. Soc. B.

[8] Dietz KJ (2015). Efficient high light acclimation involves rapid processes at multiple mechanistic levels. J. Exp. Bot..

[9] Savary S (2019). The global burden of pathogens and pests on major food crops. Nat. Ecol. Evol..

[10] de Torres Zabala M (2015). Chloroplasts play a central role in plant defence and are targeted by pathogen effectors. Nat. Plants.

[11] Gao C (2020). Pathogen manipulation of chloroplast function triggers a light-dependent immune recognition. Proc. Natl Acad. Sci. USA.

[12] Rodriguez-Herva JJ (2012). A bacterial cysteine protease effector protein interferes with photosynthesis to suppress plant innate immune responses. Cell Microbiol..

[13] Smith NA, Eamens AL, Wang MB (2011). Viral small interfering RNAs target host genes to mediate disease symptoms in plants. PLoS Pathog..

[14] Yu G (2020). A bacterial effector protein prevents MAPK-mediated phosphorylation of SGT1 to suppress plant immunity. PLoS Pathog..

[15] Nekrasov V (2017). Rapid generation of a transgene-free powdery mildew resistant tomato by genome deletion. Sci. Rep..

[16] Blanco NE (2013). Expression of the minor isoform pea ferredoxin in tobacco alters photosynthetic electron partitioning and enhances cyclic electron flow. Plant Physiol..

[17] Hussey BJ, McMillen DR (2018). Programmable T7-based synthetic transcription factors. Nucleic Acids Res..

[18] McBride KE, Schaaf DJ, Daley M, Stalker DM (1994). Controlled expression of plastid transgenes in plants based on a nuclear DNA-encoded and plastid-targeted T7 RNA polymerase. Proc. Natl Acad. Sci. USA.

[19] Yin Z, Machius M, Nestler EJ, Rudenko G (2017). Activator Protein-1: redox switch controlling structure and DNA-binding. Nucleic Acids Res..

[20] Sicilia MNA, Romero MES, Rosales MPR, Venema K (2021). Plastidial transporters KEA1 and KEA2 at the inner envelope membrane adjust stromal pH in the dark. N. Phytol..

[21] Mazzei L, Cianci M, Benini S, Ciurli S (2019). The impact of pH on catalytically critical protein conformational changes: the case of the urease, a nickel enzyme. Chemistry.

[22] Hall TM (2016). De-coding and re-coding RNA recognition by PUF and PPR repeat proteins. Curr. Opin. Struct. Biol..

[23] Bally J (2009). Plant physiological adaptations to the massive foreign protein synthesis occurring in recombinant chloroplasts. Plant Physiol..

[24] Lentz EM (2010). High expression level of a foot and mouth disease virus epitope in tobacco transplastomic plants. Planta.

[25] Occhialini, A. et al. Mini-synplastomes for plastid genetic engineering. *Plant Biotechnol. J.*10.1111/pbi.13717 (2021). 10.1111/pbi.13717 PMC8753362 34585834

[26] Chen JH (2020). Nuclear-encoded synthesis of the D1 subunit of photosystem II increases photosynthetic efficiency and crop yield. Nat. Plants.

[27] Saxena B (2014). Metabolic engineering of chloroplasts for artemisinic acid biosynthesis and impact on plant growth. J. Biosci..

[28] Llorente B (2020). Synthetic conversion of leaf chloroplasts into carotenoid-rich plastids reveals mechanistic basis of natural chromoplast development. Proc. Natl Acad. Sci. USA.

[29] Llorente B, Williams TC, Goold HD (2018). The multiplanetary future of plant synthetic biology. Genes.

[30] Wurtzel ET (2019). Revolutionizing agriculture with synthetic biology. Nat. Plants.

